# Supplementary material for: Taxonomic, biological and geographical traits of species in a coastal dune flora in the southeastern Cape Floristic Region: regional and global comparisons
Source: PeerJ. 2019 Jul 31;7:e7336. doi: 10.7717/peerj.7336 (PMC6679649; doi:10.7717/peerj.7336)
Supplement: Supplemental Information 4 — Conservation status and threats as per the Red List of South African Plants Online v. 2017.1 (http://redlist.sanbi.org). [file peerj-07-7336-s004.docx]

**Table S1:**

**Species of conservation concern in the Cape St Francis dune flora from the south-eastern Cape Floristic Region. Conservation status and threats as per the Red List of South African Plants Online v. 2017.1 (http://redlist.sanbi.org).**

| **Family** | **Species** | **Conservation status** | **Threats** |
| --- | --- | --- | --- |
| Threatened species: | | | |
| Amaryllidaceae | *Brunsvigia litoralis* | Endangered | Coastal development, alien plant invasion |
| Asteraceae | *Othonna rufibarbis* | Vulnerable | Coastal development, alien plant invasion, agriculture |
| Asteraceae | *Syncarpha sordescens* | Vulnerable | Coastal development, alien plant invasion |
| Brassicaceae | *Heliophila linearis* var. *reticulata* | Vulnerable | Coastal development, alien plant invasion |
| Dioscoreaceae | *Dioscorea sylvatica* | Vulnerable | Overharvesting (medicinal) |
| Ericaceae | *Erica chloroloma* | Vulnerable | Coastal development, alien plant invasion |
| Ericaceae | *Erica glumiflora* | Vulnerable | Coastal development, alien plant invasion |
| Fabaceae | *Aspalathus recurvispina* | Critically Endangered | Coastal development, alien plant invasion |
| Geraniaceae | *Pelargonium suburbanum* subsp. *suburbanum* | Vulnerable | Coastal development, alien plant invasion, agriculture |
| Iridaceae | *Gladiolus huttonii* | Vulnerable | Coastal development, alien plant invasion, agriculture |
| Myrsinaceae | *Rapanea gilliana* | Endangered | Coastal development, alien plant invasion |
| Orchidaceae | *Satyrium hallackii* subsp. *hallackii* | Endangered | Coastal development, alien plant invasion |
| Orchidaceae | *Satyrium princeps* | Vulnerable | Coastal development, alien plant invasion |
| Orobanchaceae | *Hyobanche robusta* | Endangered | Coastal development, alien plant invasion |
| Poaceae | *Capeochloa cincta* subsp. *sericea* | Vulnerable | Coastal development, alien plant invasion |
| Rutaceae | *Agathosma stenopetala* | Vulnerable | Coastal development, alien plant invasion |
| Other species of conservation concern: | | | |
| Aizoaceae | *Delosperma saxicola* | Rare |  |
| Amaryllidaceae | *Boophone disticha* | Declining |  |
| Amaryllidaceae | *Eucomis autumnalis* | Declining |  |
| Apiaceae | *Centella tridentata* var. *hermanniifolia* | Rare |  |
| Asteraceae | *Arctotis elongata* | Data deficient |  |
| Asteraceae | *Seriphium* sp. nov. “dunensis” | Data deficient |  |
| Cyperaceae | *Schoenus* sp. nov. “cuspidata dunes” | Data deficient |  |
| Cytinaceae | *Cytinus* sp. nov. | Data deficient |  |
| Fabaceae | *Indigofera tomentosa* | Near Threatened |  |
| Fabaceae | *Psoralea repens* | Near Threatened |  |
| Iridaceae | *Moraea australis* | Near Threatened |  |
| Myrsinaceae | *Rapanea melanophloeos* | Declining |  |
| Orchidaceae | *Eulophia speciosa* | Declining |  |
| Plumbaginaceae | *Limonium* sp. nov. “St Francis” | Data deficient |  |
| Scrophulariaceae | *Nemesia fourcadei* | Data deficient |  |
