# Supplementary material for: Taxonomic, biological and geographical traits of species in a coastal dune flora in the southeastern Cape Floristic Region: regional and global comparisons
Source: PeerJ. 2019 Jul 31;7:e7336. doi: 10.7717/peerj.7336 (PMC6679649; doi:10.7717/peerj.7336)
Supplement: Supplemental Information 5 [file peerj-07-7336-s005.docx]

**Table S2:**

**Plant species-to-genus ratios for Holocene coastal dune sites from the Cape Floristic Region and other Mediterranean-Climate Ecosystems.**

| **Site** | **Species:genus ratio** | **Source** |
| --- | --- | --- |
| Cape Floristic Region: | | |
| Table Bay | 2.11 | Supplemental Data S2 (cf Low, 2011) |
| Walker Bay | 1.81 | Supplemental Data S3 (cf Privett and Lutzeyer, 2010) |
| Struisbaai | 1.60 | Zietsman (2003) |
| Cape St Francis | 1.50 | This study |
| California: | | |
| Guadalupe–Nipomo | 1.4 | US Fish & Wildlife Service (2016) |
| Mediterranean Basin: | | |
| Tuscany | 1.67 | Ciccarelli et al. (2014) |
| El Rompido spit, southern Spain | 1.29 | Muñoz Vallés et al. (2009) |
| Cyprus | 1.62 | Hadjichambis et al. (2004) |
| Evrotas Delta, southern Greece | 1.24 | Korakis and Gerasimidis (2006) |
| West coast, Greece | 1.42 | Spanou et al. (2006) |
| Western Australia | | |
| Jurien Bay | 1.71 | Zemunik (2016) |

**References**

Ciccarelli, D., Di Bugno, C. and Peruzzi, L. 2014. Checklist della flora vascolare psammofila della Toscana. *Atti della Società Toscana di Scienze Naturali - Memorie Serie B* 121: 37-88. DOI: 10.2424/ASTSN.M.2014.05.

Hadjichambis, A.C., Della, A., Paraskeva-Hadjichambi, D., Georghiou, K. and Dimopoulos, P. 2004. Flora of the sand dune ecosystems of Cyprus. In: Arianoutsou and Papanastasis (eds). *Proceedings of the 10^th^ MEDECOS Conference*, 25 April – 1 May 2004, Rhodes, Greece. Millpress, Rotterdam. Available at http://users.uoa.gr/~kgeorghi/files/7_flora_of_the_sand_dune_ecosystems_of_cyprus.pdf (accessed 29 March 2018).

Korakis, G. and Gerasimidis, A. 2006. Coastal and halophytic habitats and their flora in Evrotas Delta (SE Peloponnisos, Greece). *Journal of Biological Research* 6: 155-166.

Low, A.B. 2011. *Botanical and Dune Ecology Impact Assessment for the Proposed Nuclear 1, 2 and 3 Sites at Koeberg (Duynefontein), Bantamsklip and Thyspunt*. Coastal and Environmental Consultants, Rondebosch, Cape Town.

Muñoz Vallés, S., Gallego Fernández, J.B. and Dellafiore, C. 2009. Estudio florístico de la Flecha Litoral de El Rompido (Lepe, Huelva) Análisis y catálogo de la flora vascular de los sistemas de duna y marisma. *Lagascalia* 29: 43-88.

Privett, S. and Lutzeyer, H. 2010. *Field Guide to the Flora of Grootbos Nature Reserve and the Walker Bay Region*. Grootbos Foundation, Hermanus.

Spanou, S., Verroios, G., Dimitrellos, G., Tiniakou, A. and Georgiadis, T. 2006. Notes on flora and vegetation of the sand dunes of western Greece. *Willdenowia* 36: 235-246.

US Fish & Wildlife Service. 2016. *Guadalupe-Nipomo Dunes National Wildlife Refuge. Final Comprehensive Conservation Plan and Environmental Assessment.* Available at https://www.federalregister.gov/documents/2016/03/02/2016-04571/guadalupe-nipomo-dunes-national-wildlife-refuge-san-luis-obispo-county-ca-draft-comprehensive (accessed 23 March 2018).

Zemunik, G.A.Z. 2016. *Jurien Bay Dune Chronosequence Floristics and Soil Dataset, Version 1.0*. http://doi.org/10.4227/05/56AEB32FC11D4. ÆKOS Data Portal, rights owned by University of Western Australia.

Zietsman, M.M. 2003. *Phytosociological study of Andrew’s Field and Tsaba-Tsaba Nature Reserve, Bedasdorp District, Western Cape*. Masters thesis, Department of Botany, Faculty of natural and Agricultural Sciences, University of Pretoria, Pretoria.
